# Factors associated with changes in illicit opioid use during the COVID-19 pandemic among incarcerated people who use drugs in Quebec, Canada

**DOI:** 10.1108/IJPH-06-2022-0038

**Published:** 2022-12-19

**Authors:** Hyejin Park, Blake Linthwaite, Camille Dussault, Alexandros Halavrezos, Sylvie Chalifoux, Jessica Sherman, Lina Del Balso, Jane A. Buxton, Joseph Cox, Nadine Kronfli

**Affiliations:** Research Institute of the McGill University Health Centre, Montreal, Canada; University of British Columbia, Vancouver, Canada; Research Institute of the McGill University Health Centre, Montreal, Canada; McGill University, Montreal, Canada and McGill University Health Centre, Montreal, Canada; Research Institute of the McGill University Health Centre, Montreal, Canada; McGill University, Montreal, Canada and McGill University Health Centre, Montreal, Canada

**Keywords:** Prison, Incarceration, Opioids, Drug use, SARS-CoV-2, COVID-19 pandemic

## Abstract

**Purpose:**

People who use drugs (PWUD) have been disproportionately affected by the COVID-19 pandemic. This study aims to examine changes in illicit opioid use and related factors among incarcerated PWUD in Quebec, Canada, during the pandemic.

**Design/methodology/approach:**

The authors conducted an observational, cross-sectional study in three Quebec provincial prisons. Participants completed self-administered questionnaires. The primary outcome, “changes in illicit opioid consumption,” was measured using the question “Has your consumption of opioid drugs that were not prescribed to you by a medical professional changed since March 2020?” The association of independent variables and recent changes (past six months) in opioid consumption were examined using mixed-effects Poisson regression models with robust standard errors. Crude and adjusted risk ratios with 95% confidence intervals (95% CIs) were calculated.

**Findings:**

A total of 123 participants (median age 37, 76% White) were included from January 19 to September 15, 2021. The majority (72; 59%) reported decreased illicit opioid consumption since March 2020. Individuals over 40 were 11% less likely (95% CI 14–8 vs 18–39) to report a decrease, while those living with others and with a history of opioid overdose were 30% (95% CI 9–55 vs living alone) and 9% (95% CI 0–18 vs not) more likely to report decreased illicit opioid consumption since March 2020, respectively.

**Originality/value:**

The authors identified possible factors associated with changes in illicit opioid consumption among incarcerated PWUD in Quebec. Irrespective of opioid consumption patterns, increased access to opioid agonist therapy and enhanced discharge planning for incarcerated PWUD are recommended to mitigate the harms from opioids and other drugs.

## Introduction

People who use drugs (PWUD) have been disproportionately affected by the COVID-19 pandemic (Minoyan *et al.*, 2022). Substance use disorders are common among PWUD, and changes in consumption have resulted in a 95% increase in opioid toxicity deaths in the first year of the COVID-19 pandemic in Canada (Government of Canada, 2022). Several factors may have contributed, including disruptions to harm reduction services, lockdown mandates resulting in altered coping strategies and changes to the drug market secondary to travel restrictions (Canadian Centre on Substance Use and Addiction, 2020). Because of the intersection of illicit substance use and incarceration, individuals with substance use disorders are often incarcerated; however, the effect of the COVID-19 pandemic on opioid consumption among incarcerated PWUD is poorly understood. Service disruptions related to opioid agonist therapy (OAT) and reduced drug availability because of suspended visits and supply disruptions suggest that PWUD may have had reduced access to illicit drugs in carceral settings [European Monitoring Centre for Drugs and Drug Addiction (EMCDDA), 2021].

Very few studies have thus far sought to understand predictors of changes in illicit opioid consumption during the COVID-19 pandemic. Those studies have found that age (Friesen *et al.*, 2021), ethnicity (Friesen *et al.*, 2021), unstable employment (Ali *et al.*, 2021; Rosic *et al.*, 2021), unstable housing (Friesen *et al.*, 2021; Minoyan *et al.*, 2022; Rosic *et al.*, 2021) and social isolation (Friesen *et al.*, 2021) were associated with changes in opioid consumption. However, none of these studies were conducted among incarcerated PWUD. Understanding factors related to changes in illicit opioid use among incarcerated PWUD is crucial in developing strategies to address those who may be at the highest risk of overdose and death while incarcerated or once released. We aimed to determine factors associated with changes in illicit opioid use among incarcerated PWUD in Quebec, Canada, during the COVID-19 pandemic.

## Methods

### Study design and setting

We conducted an observational cross-sectional study in three Quebec provincial prisons, where individuals are sentenced for less than two years, representing 45% of the incarcerated male provincial population in Quebec (Gouvernement du Québec, 2019). The study sites included l’Établissement de détention de Montréal (EDM), l’Établissement de détention de Rivière-des-Prairies (EDRDP) and l’Établissement de détention de St-Jérôme (EDSJ).

### Participants

We included incarcerated men aged 18 years or older who were able to consent to study participation in either English or French. We excluded those in isolation with active SARS-CoV-2, under investigation for COVID-19, and those who posed a security risk to the research team. Participants provided written informed consent and received an honorarium of $10 CAD for study participation. This study was approved by the McGill University Health Centre Research Ethics Board (#2021–6888) and the Direction régionale des services correctionnels du Québec (#2020–12493).

This study represents secondary use of data collected for a SARS-CoV-2 serological study conducted at the study sites (Kronfli *et al.*, 2022).

### Data collection

Convenience sampling of individuals meeting the eligibility criteria was undertaken. Incarcerated individuals were approached in their cells by the research team. Participants who agreed to participate were given self-administered questionnaires that included questions on sociodemographic and clinical characteristics, as well as opioid consumption.

### Outcome measure and variables of interest

We hypothesized that illicit opioid use among incarcerated PWUD would decrease during the COVID-19 pandemic primarily because of supply disruptions. The primary outcome measure, “changes in illicit opioid consumption,” was dichotomized into decreased vs increased/same. This was measured by participants’ responses to the question “Has your consumption of opioid drugs that were not prescribed to you by a medical professional changed since March 2020?” choosing among “Yes, decreased,” “Yes, increased,” “No, stayed the same” and “Prefer not to answer.”

Independent variables were determined *a priori*, based on a literature review of factors associated with opioid consumption patterns among PWUD during the COVID-19 pandemic. These included age (Friesen *et al.*, 2021), ethnicity (Friesen *et al.*, 2021), income (Ali *et al.*, 2021; Rosic *et al.*, 2021), household density (Friesen *et al.*, 2021; Rosic *et al.*, 2021) and history of overdose (Flam-Ross *et al.*, 2022). Unstable housing was also associated with changes in opioid consumption (Friesen *et al.*, 2021; Rosic *et al.*, 2021) but was excluded because of its correlation with income.

### Statistical analyses

Summary statistics, medians and interquartile ranges (IQRs) for continuous variables and counts and proportions for categorical variables were calculated to describe the study sample. Self-reported ethnicity was dichotomized into White vs other (i.e. Indigenous, Black, Latin American, Arab, Asian). Annual income, household density and history of overdose were measured by participants’ responses to the questions: “Last year, what was your total annual income from all paid work and all other sources before taxes and other deductions?” ($0–$29,999 CAD vs ≥$30,000 CAD), “Since March 2020, how many people were living with you?” and “Have you overdosed (on purpose or by accident) on an opioid drug since March 2020?” (yes vs no), respectively. March 2020 corresponded to the start of the SARS-CoV-2 pandemic in Quebec, Canada.

Mixed-effects Poisson regression models with robust standard errors were used to examine correlates associated with changes in opioid consumption since the start of the COVID-19 pandemic among people who reported recent opioid consumption – that is, within the last six months – to capture the true effect of the COVID-19 pandemic. We calculated crude and adjusted risk ratios (aRR) with 95% confidence intervals (95% CIs) as they are more easily interpretable than odds ratios provided by logistic regression (Knol *et al.*, 2012). To account for the variability between correctional facilities, the three study sites were included as random intercepts. Participants who selected “Prefer not to answer” for the primary outcome measure were removed from regression analyses (*n* = 15). Multiple imputation was performed for any other missing values. All analyses were performed in R statistical software (version 4.1.2), using the “GLMMadaptive” package for the mixed-effect models and the “mice” package for multiple imputation.

## Findings

### Sample characteristics

**Figure 1:**
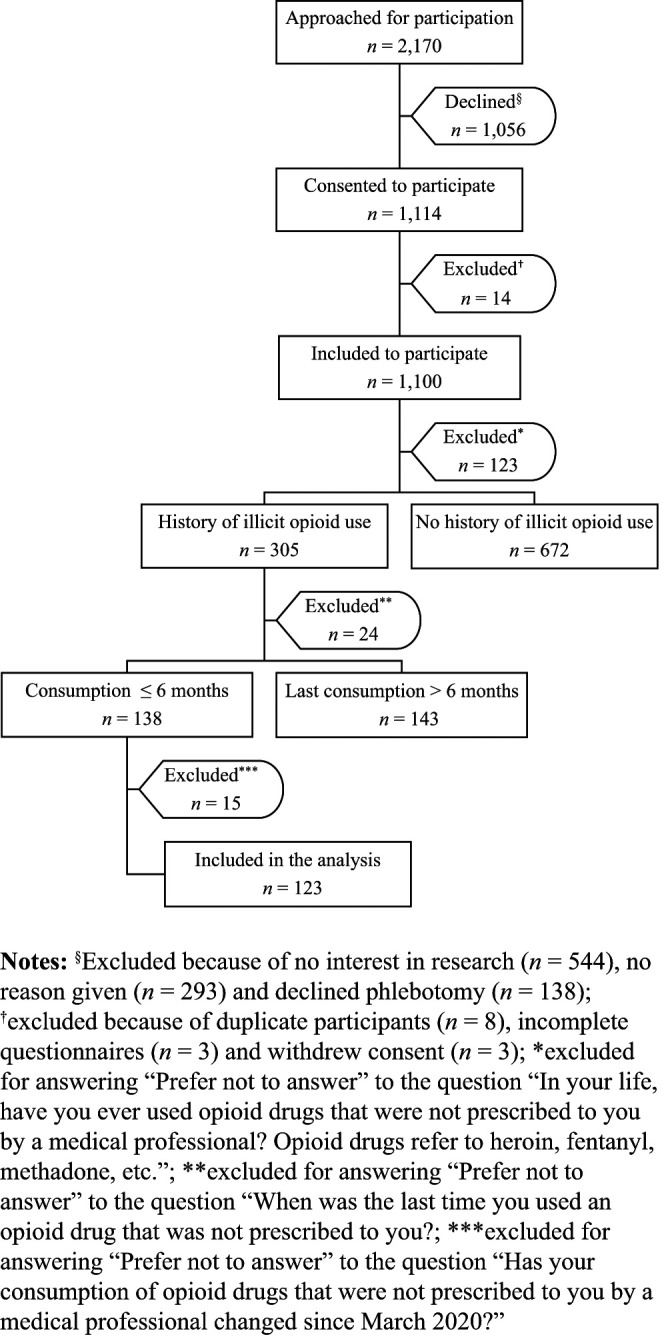
Sample selection flow chart of study participants at the three provincial prisons in Quebec, Canada

A total of 2,170 incarcerated individuals across the three provincial prisons were invited to participate (*n* = 1,181 at EDM, *n* = 549 at EDRDP and *n* = 440 at EDSJ) (Figure 1). Of these, 1,056 (49%) declined participation, more than half of whom were not interested in participating in research. An additional 14 participants were excluded, leaving 1,100 participants who agreed to participate from January 19 to September 15, 2021 (*n* = 600 at EDM, *n* = 300 at EDRDP and *n* = 200 at EDSJ), of whom 305 (28%) reported a history of illicit opioid use. Of these, 281 answered the question related to timing of most recent consumption; 138 (49%) reported use within the last six months (which represented opioid use between July 2020 and March 2021). Among these, 15 participants were excluded as they declined to answer the question related to our primary outcome (i.e. changes in opioid consumption), leaving 123 participants in the analysis (*n* = 63 at EDM, *n* = 45 at EDRDP and *n* = 15 at EDSJ).

Sociodemographic characteristics are reported in Table 1. Overall, the median age was 37 years. Three-quarters (76%) self-identified as White. Half (50%) reported a personal gross yearly income of less than $30,000 CAD and 60% reported living with at least one other person prior to incarceration. Approximately one-quarter (23%) reported a history of opioid overdose since March 2020. Over one-third (35%) spent more than 50% of their time in a Quebec provincial prison since March 2020, while 26% were incarcerated for less than four weeks (data not shown).

### Illicit opioid consumption changes among individuals with recent use since March 2020

The majority (72/123; 59%) of participants reported a decrease in illicit opioid consumption since March 2020. Of these, 18 (25%) reported a history of overdose since March 2020. Conversely, a total of 51 (41%) participants reported increased (*n* = 24) or unchanged (*n* = 27) consumption. Of these, 10 (20%) reported a history of overdose since March 2020.

Factors associated with decreased consumption of illicit opioids identified in univariate and multivariable analyses are presented in Table 2. In the univariate model, decreased consumption of illicit opioids was significantly associated with age over 40, income ≥$30,000 CAD, household density ≥ 2 and a history of opioid overdose since the start of the COVID-19 pandemic. In the multivariable model, individuals over 40 were 11% less likely (95% CI 14–8% vs 18–39) to report a decrease in illicit opioid consumption while those living with others and with a history of opioid overdose since March 2020 were 30% (95% CI 9–55% vs living alone) and 9% (95% CI 0–18% vs not) more likely to report a decrease in illicit opioid consumption since the start of the COVID-19 pandemic, respectively.

## Discussion

Our cross-sectional study explored reported changes in opioid use and factors associated with changes in illicit opioid consumption among incarcerated PWUD during the COVID-19 pandemic. Our study found that approximately 60% of incarcerated PWUD reported decreased opioid use since the start of the pandemic, in keeping with what has been reported in European prisons (EMCDDA, 2021). We also found that those who reported living with others or with a history of overdose were more likely to decrease illicit opioid consumption during the COVID-19 pandemic, a finding that has important consequences on public health policies in prisons and surrounding communities.

Decreased opioid consumption may have been the result of reduced opioid availability, concerns regarding the risk of (repeat) overdose and knowledge of an increasingly toxic drug supply during the pandemic (Gouvernement du Québec, 2022). Furthermore, to mitigate some of the expected consequences of the COVID-19 pandemic on PWUD, Canadian health authorities expedited modifications to existing services; for example, increased allowances for take-home dosing of OAT were permitted (Government of Canada, 2021) and may in part explain our findings. While decreased opioid consumption appears encouraging, as the pandemic has progressed, prisons worldwide have witnessed an increase in alternative supply routes, including the use of drones, and increased availability of alternate drugs such as benzodiazepines (EMCDDA, 2021), resulting in substantial changes in the substances used by PWUD.

The COVID-19 pandemic has not only impacted access to illicit drugs in correctional settings but has also had differing and evolving effects on access to OAT services for incarcerated PWUD. For example, in the USA, early in the pandemic, there was reduced access to OAT programs in many correctional institutions (Bandara *et al.*, 2020) – a finding that was mirrored in many European prisons because of interruptions in the provision of OAT (Shirley-Beavan, 2021; Montanari *et al.*, 2021). However, the availability of OAT appears to have increased in US jails and prisons during the pandemic (Dadiomov *et al.*, 2022), likely in part because of the relaxation of regulations that allowed increased prescribing of OAT. As of January 1, 2020, requirements mandating in-person physical evaluations of patients treated with buprenorphine were lifted in the USA (Substance Abuse and Mental Health Services Administration, 2020), including in correctional settings. This led to the start of remote (i.e. via telehealth) buprenorphine initiations in US jails (Duncan *et al.*, 2021) and other “technology-infused” treatment innovations (Donelan *et al.*, 2021). Furthermore, because of increased and expedited decarceration during the pandemic, comprehensive discharge planning, and in particular, for those on OAT, became even more critical. Barriers to OAT retention following release (Russell *et al.*, 2022b; Russell *et al.*, 2022c) were heightened during the pandemic because of scarcer community services for PWUD (Minoyan *et al.*, 2022; Söderholm, 2021), underscoring the need to strengthen correctional and community partnerships and to develop specific re-entry protocols to facilitate linkage to OAT programs (Zaller and Brinkley-Rubinstein, 2020). In fact, some correctional settings experimented with take-home OAT (“carries”) at release to facilitate continuity of care (Duncan *et al.*, 2021; Russell *et al.*, 2022a) while case reports emerged for the use of low-threshold telemedicine for the provision of buprenorphine–naloxone following release (Flavin *et al.*, 2022). Peer health mentorship was also found to be key for community re-entry during the pandemic (McLeod *et al.*, 2021) and should be explored going forward.

The impact of mandatory quarantine following prison admission or repetitive preventative isolation during incarceration on mental health, illicit drug use and associated deaths has yet to be fully explored (Johnson *et al.*, 2021). The heightened risk of COVID-19, combined with suboptimal prison conditions, has been found to adversely impact the mental health of incarcerated PWUD, resulting in increased substance use (Mental Health Commission of Canada, 2021). A total of 20% of study participants reported increased opioid use during the pandemic. In fact, Quebec provincial prisons witnessed the highest recorded number of attempted suicides in 2020/2021, with twice the number of suicides compared with 2019/2020 (Ministère de la Sécurité publique, 2021), consistent with other countries (Gétaz *et al.*, 2021). Isolation has also been found to be associated with increased opioid-related harms (Friesen *et al.*, 2021), and opioid-related deaths in Canada have burgeoned during the pandemic, driven primarily by increases in fentanyl and its analogues in the drug supply (Gouvernement du Québec, 2022; Government of Canada, 2022). Furthermore, there has been increased demand for OAT among incarcerated PWUD who underwent quarantine (EMCDDA, 2021). That said, we found that living with others is protective against increased opioid consumption. These findings further advocate for the need for increased access to OAT and other harm reduction services in prison and at the time of release, discharge planning to shared living environments (cognizant of the increased risk of SARS-CoV-2 acquisition during peak periods of community transmission) and decarceration as a mitigation strategy against mental health and substance use.

Our study has limitations. First, the study sample was restricted to adult men incarcerated in 3 of 16 Quebec provincial prisons, reducing the generalizability of our study results. Second, the questionnaire was designed to answer an alternative primary outcome (Kronfli *et al.*, 2022) and thus lacked detailed questions on substances used, access to OAT, drug treatment programs, harm reduction services and reasons for changes in substance use. Third, the findings of our study are not restricted to changes in illicit opioid use during incarceration. The Canadian provincial prison population has both a high rate of turnover and recidivism such that findings may represent changes to illicit opioid consumption among PWUD in the community and/or during incarceration. However, a substantial proportion (35%) of our study population spent more than 50% of their time in provincial prison since March 2020. Fourth, information related to illicit opioid consumption was collected from study questionnaires during incarceration, which may have introduced response biases (acquiescence, social desirability and dissent). However, questionnaires were self-administered and collected by the research team to maintain confidentiality, which may have mitigated these biases. Lastly, our sample size was small because of the exclusion of many participants who failed to respond to several questions (Figure 1). Given the small number of participants who reported increased (*n* = 24) or unchanged (*n* = 27) opioid consumption, we were not powered to determine factors associated with each of these changes. Additional and larger studies focused exclusively on illicit drug use during the COVID-19 among incarcerated PWUD are thus needed. Despite these limitations, our study adds to the dearth of data evaluating the impact of COVID-19 on illicit opioid consumption among this vulnerable population.

## Conclusion

We identified possible factors associated with changes in illicit opioid consumption among incarcerated PWUD in Quebec, Canada. Irrespective of the pattern of opioid consumption, increased access to OAT and enhanced discharge planning are required to mitigate the harms from opioids or other drugs for this population.

## Acknowledgements

The authors thank all of the study participants, correctional agents as well as the Directors of Professional Services at the three provincial prisons who granted access to the research team during the COVID-19 pandemic.

*Funding*: This work was supported by the Public Health Agency of Canada through the Sero-Surveillance and Research (COVID-19 Immunity Task Force Initiative) Program (2021-HQ-000103). The funder had no role in study design, data collection and analysis, decision to publish or preparation of the manuscript. NK is supported by a career award from the Fonds de Recherche Québec–Santé (FRQ-S; junior 1).

*Declarations of interest*: HP, BL, CD, AH, SC, JS, LDB, JB report no potential conflicts. JC has received research funding from ViiV Healthcare for an investigator-initiated study and from Gilead Sciences for clinical trials and reports remuneration for advisory work (ViiV Healthcare, Gilead Sciences, and Merck Canada) outside of the submitted work. NK reports research funding from Gilead Sciences, McGill Interdisciplinary Initiative in Infection and Immunity, Canadian Institutes of Health Research and Canadian Network on Hepatitis C; reports advisory fees from Gilead Sciences, ViiV Healthcare, Merck and AbbVie; and reports speaker fees from Gilead Sciences, AbbVie and Merck, all outside of the submitted work. All authors have submitted the declaration of interest statement.

*Author contributions*: HP: Conceptualization, Methodology, Software, Formal analysis, Investigation, Data curation, Writing – original draft, Writing – review & editing, Visualization, Project administration. BL: Methodology, Software, Formal analysis, Writing – review & editing. CD: Conceptualization, Methodology, Formal analysis, Data curation, Writing – review & editing, Project administration. AH: Investigation, Writing – review & editing, Project administration. SC: Investigation, Writing – review & editing. JS: Investigation, Writing – review & editing. LDB: Investigation, Writing – review & editing. JB: Writing – review & editing. JC: Conceptualization, Methodology, Writing – review & editing. NK: Conceptualization, Methodology, Formal analysis, Investigation, Writing – original draft, Writing – review & editing, Visualization, Supervision, Funding acquisition.

*Ethics approval*: The McGill University Health Centre Research Ethics Board (#2021-6888) and the Direction régionale des services correctionnels du Québec approved the study (#2020-12493).

## Figures and Tables

**Table 1:** Baseline characteristics and changes in consumption of illicit opioid drugs among adults incarcerated in Quebec, Canada

Characteristic	Decreased n = 72	Increased/unchanged n = 51	Total N = 123
*Age (median [IQR])*	36 [18, 58]	38 [19, 72]	37 [18, 72]
*Age category*			
18–39 years	46 (64%)	28 (55%)	74 (60%)
≥40 years	26 (36%)	23 (45%)	49 (40%)
*Ethnicity*			
White	54 (75%)	40 (78%)	94 (76%)
Other^§^	18 (25%)	11 (22%)	29 (24%)
*Annual income (in CAD)*			
$0–$29,999	37 (51%)	24 (47%)	61 (50%)
≥$30,000	22 (31%)	21 (41%)	43 (35%)
Missing	13 (18%)	6 (12%)	19 (15%)
*Household density* ^¶^^†^			
1 (i.e. living alone)	21 (29%)	24 (47%)	45 (37%)
≥2	48 (67%)	26 (51%)	74 (60%)
Missing	3 (4%)	1 (2%)	4 (3%)
*History of opioid overdose since March 2020*			
No	52 (72%)	40 (78%)	92 (75%)
Yes	18 (25%)	10 (20%)	28 (23%)
Missing	2 (3%)	1 (2%)	3 (2%)
*Correctional facility*			
EDRDP	26 (36%)	19 (37%)	45 (37%)
EDM	37 (51%)	26 (51%)	63 (51%)
EDSJ	9 (13%)	6 (12%)	15 (12%)

Notes: CAD: Canadian dollars; EDM: l’Établissement de détention de Montréal; EDSJ: l’Établissement de détention de St-Jérôme; EDRDP: l’Établissement de détention de Rivière-des-Prairies; IQR: interquartile range; ^§^Other includes Indigenous, Black, Latin American, Arab and Asian; ^¶^outside prison

**Table 2:** Unadjusted and adjusted associations between multiple variables of interest and decreased consumption of illicit opioid drugs among adults incarcerated in Quebec, Canada

Characteristic	Unadjusted RR (95% CI)	Adjusted* RR (95% CI)
*Age category*		
18–39 years	*Reference*	*Reference*
≥ 40 years	0.85 (0.78–0.93)	0.89 (0.86–0.92)
*Ethnicity*		
White	*Reference*	*Reference*
Other^§^	1.08 (0.95–1.23)	1.02 (0.93–1.11)
*Annual income (in CAD)*		
$0–$29,999	*Reference*	*Reference*
≥$30,000	0.80 (0.70–0.91)	0.79 (0.60–1.03)
*Household density* ^¶^		
1 (i.e. living alone)	*Reference*	*Reference*
≥2	1.32 (1.22–1.42)	1.30 (1.09–1.55)
*History of opioid overdose since March 2020*		
No	*Reference*	*Reference*
Yes	1.13 (1.04–1.23)	1.09 (1.00–1.18)

Notes: CAD: Canadian dollars; CI: confidence interval; RR: risk ratio; *adjusted for age, ethnicity, income, living alone and overdose history; ^§^Other includes Indigenous, Black, Latin American, Arab and Asian; ^¶^outside prison
